# Anticoagulant and Anti-thrombotic Treatments in the Management of Hematological Malignancies in a Home Care Program

**DOI:** 10.4103/0973-1075.78450

**Published:** 2011

**Authors:** Andrea Tendas, Luca Cupelli, Laura Scaramucci, Massimiliano Palombi, Malgorzata Monika Trawinska, Marco Giovannini, Gregorio Antonio Brunetti, Claudio Cartoni, Francesco Bondanini, Paolo de Fabritiis, Pasquale Niscola

**Affiliations:** Hematology Unit, S. Eugenio Hospital, Rome, Italy; 1 Hematology, Department of Cellular Biotechnologies and Hematology, University “La Sapienza”, Rome, Italy; 2 Laboratory Medicine, “Sandro Pertini” Hospital, Rome, Italy

**Keywords:** Acenocumarine, Bleeding, Hematological malignancies, Low molecular weight heparin, Warfarin

## Abstract

**Aim:**

Anticoagulants (AC) and anti-platelet (AP) agents are widely administered to patients with hematological malignancies (HM). However, HM patients may be at high risk of bleeding and hemorrhagic complications, because of different form of coagulopathies and several degrees of thrombocytopenia.

**Materials and Methods:**

A prospective evaluation of the use of anticoagulant and anti-thrombotic agents as well as of bleeding and thrombotic complications in a consecutive cohort of patients, which were followed during the first semester of 2010 by our home care service, was performed. In this regard, three pharmacological class of agents, such as oral anticoagulants (warfarin and acenocumarine), low molecular weight heparin (LMWH) and anti-platelet (AP) drugs were considered.

**Results:**

Out of 129 patients, 26 (20%) were treated with AC/AP drugs. Warfarin, acenocumarine, LMWH as well as AP were used in 7, 11 and 12 patients, respectively. Adverse events (bleeding) were observed in 3 patients (11.5%), 2 cases being on warfarin (replaced by LMWH) and 1 being AP (suspension without replacement); out of the 3 patients with bleeding, none presented thrombocytopenia.

**Conclusions:**

Despite the frequent findings of hemostatic disorders in a population of frail patients managed in a home care setting, our experience demonstrated that the use of AC/AP drugs has been very rarely responsible for significant complications.

## INTRODUCTION

Anticoagulant (AC) and antiplatelet (AP) agents are widely used to manage thrombotic complications, which may be frequently observed in patients with hematological malignancies (HM).[1–4] However, patients with HM may be at high risk of hemorrhagic complications due to different form of coagulopathies, several degrees of thrombocytopenia, and associated comorbidities.[5,6] So that, the safe administration of these agents in this setting represents a matter of debate, also in the light of the paucity of data regarding this issue available in the medical literature. In this regard, we have performed a single-center prospective survey on a series of consecutive patients with HM in advanced phase of disease followed in a home care setting.

## MATERIALS AND METHODS

A prospective evaluation of the use of anticoagulant and anti-thrombotic agents, as well as the bleeding and thrombotic complications in a consecutive cohort of patients followed at home during the first semester of 2010 was performed. Three pharmacological class of agents, such as oral AC (warfarin and acenocumarine), low molecular weight heparin (LMWH) and AP drugs were considered. The indication for treatment, along with any significant adverse effects potentially referable to these agents, was evaluated. All treatments were given at home, where patients were followed by a specialized and multidisciplinary trained staff, composing seven hematologists, ten nurses, and several other care providers trained in hematology, palliative care and rehabilitation medicine.[7–10] The availability at home of a reliable and trained caregiver was considered an essential condition to provide a home care program.[9] The home care team worked together with general services and diagnostic structures of our hospital; in the case of bleeding, all hemostatic measures, including platelet concentrates and fresh frozen plasma (FFP) transfusions, were supplied at home.[6]

## RESULTS

Demographic data and hematological diagnoses of patients included in the study are reported in Table 1. Out of 129 patients, 26 (20%) were treated with AC/AP drugs. Acenocumarine, warfarin, LMWH and AP were used in 7, 11 and 12 patients, respectively. Treatment indication, platelets count, coexisting renal or liver dysfunctions and drug dosage adjustment are reported on Table 2. Adverse events (bleeding) were observed in 3 (11.5%) out of 26 treated patients, being 2 of them on oral AC and 1 on AP; none of these 3 patients had thrombocytopenia.

**Table 1:** Demographic data and hematological diagnoses of patients followed during observation period

Category		*n*
Patients		129
Age (years)	78 (20-98)	
Gender	Male	57
	Female	72
Diagnoses	MDS/cMPD	53
	Acute leukemia	11
	Lymphoma	18
	Plasma cell dyscrasia	12
	Cancer-unrelated anemia	21
	Other	14
Disease status	Advanced / terminal	32
	Indolent / chronic	78
	Active treatment	19

**Table 2:** Treatment indication, platelets count, coexisting renal or liver failure, drug dosage modification in AC / AP patients

Category		*n*
Patients		26
AC / AP indication	Primary prophylaxis a	11
	Secondary prophylaxis b	14
	Treatment	1
AC / AP target	Ischemic heart disease	9
	Other heart disease	6
	DVT	6
	Ischemic cerebrovascular disease	5
Platelets count	Low (<150,000)	8
	Normal	14
	High (>450,000)	4
Renal failure	Yes	11
	No	15
Liver failure	Yes	0
	No	26
Drug adjustment	Yes for bleeding risk	2
	Yes for severe renal failure	1
	No	23

AC - anticoagulants; AP - anti-platelet

a Treatment intended to prevent the first occurrence of thrombotic event

b preventive treatment for a subsequent occurrence (relapse) of thrombotic event. DVT: deep vein thrombosis

## CONCLUSIONS

In the management of patients with HM, thrombotic complications may represent a considerable concern, especially when they are in advanced phase of their disease and are followed at home. The incidence of these potentially devastating complications in patients with HM has been reported to be higher than that observed in the setting of solid tumors. Contributing factors include a HM-related thrombophilic state, some underlying disease activities and certain antineoplastic therapies, such as high dose corticosteroids, new immunomodulatory agents and hematopoietic growth factors. Primary and secondary pharmacological prophylaxis can be problematic in these patients, who are often concerned by thrombocytopenia, coagulopathies or co-existing diseases and organ dysfunctions for which a dosage adjustment of AC and AP drugs and a careful clinical monitoring are required. Our experience is referred to the activity of a home care services implemented in Rome,[7–10] during the last two decades with the aim to offer highly developed expertise to several categories of patients with HM. In our experience, the home care represented an important added value in the global management of patients with deteriorated clinical conditions, social difficulties and physical impairments, achieving an integrated model of assistance and a cost-effective form of patient’s care.[7] Despite the frequent findings of hemostatic disorders in this population of frail patients managed in a home care setting, our experience demonstrated that AC and AP drugs have been very rarely responsible for significant complications.
